# The Ensembl Variant Effect Predictor

**DOI:** 10.1186/s13059-016-0974-4

**Published:** 2016-06-06

**Authors:** William McLaren, Laurent Gil, Sarah E. Hunt, Harpreet Singh Riat, Graham R. S. Ritchie, Anja Thormann, Paul Flicek, Fiona Cunningham

**Affiliations:** European Molecular Biology Laboratory, European Bioinformatics Institute, Wellcome Genome Campus, Hinxton, Cambridge CB10 1SD UK

**Keywords:** Variant annotation, NGS, Genome, SNP

## Abstract

The Ensembl Variant Effect Predictor is a powerful toolset for the analysis, annotation, and prioritization of genomic variants in coding and non-coding regions. It provides access to an extensive collection of genomic annotation, with a variety of interfaces to suit different requirements, and simple options for configuring and extending analysis. It is open source, free to use, and supports full reproducibility of results. The Ensembl Variant Effect Predictor can simplify and accelerate variant interpretation in a wide range of study designs.

## Background

Analysis of variant data resulting from genome or exome sequencing is fundamental for progress in biology, from basic research to translational genomics in the clinic. It is key for investigating function and for progressing from a system of medical care based on standardized treatment to one targeted to the individual patient.

For sufferers of common or rare disease, the potential benefits of variant analysis include improving patient care, surveillance, and treatment outcomes. In cancer, there have already been numerous successes using data from genetic tests. For example, patients testing positive for the inheritance of BRCA mutations have the option of selective preventative surgery; lung cancer patients showing EGFR gene mutations or triple negative breast cancer patients can have their drug prescriptions tailored to improve success [1, 2].

Rare diseases can individually be difficult to diagnose due to the low incidence and the incomplete penetrance of implicated alleles. However, variant analysis of whole-genome sequencing (WGS) or whole-exome sequencing data can lead to the discovery of underlying genetic mutations [3]. Identifying an associated mutation is advantageous for researching treatment options and for future drug discovery. Meanwhile, even the immediate benefit of diagnosis may result in a more accurate prognosis and remove the burden of additional medical investigations.

The most common non-infectious diseases worldwide are cardiovascular disease, cancer, and diabetes [4]. Despite many array-based genome-wide association studies (GWAS) searching for risk loci, only a relatively small heritable component in these conditions has been elucidated [5]. WGS in large numbers of samples is required to yield enough statistical power to detect rare variants with potential phenotypic or disease associations [6, 7]. WGS studies will also detect variants in regulatory and non-coding regions of the genome, which are thought to comprise the majority of trait-associated variants [8] and play a role in cancer [9].

The potential of large-scale sequencing and variant analysis is revolutionary. Recognizing this value, major population sequencing initiatives have been launched in Iceland [10], the UK [11], and the USA [12]. In other species, efforts such as Genome 10 K [13], the 1001 Arabidopsis genomes [14], and 1000 bull genome project [15] have similar goals but operate under different funding models, often with less support than the *Homo sapiens*-focused projects.

Ongoing improvements in DNA sequencing technology, and a current cost around $1000 per human genome, have resulted in high volumes of genome, exome, and subsequent variant data requiring interpretation. Meanwhile, the cost of the analysis to determine functional consequences remains substantially higher due to the difficulty of variant interpretation. For example, a typical diploid human genome has around 3.5 million single nucleotide variants (SNVs) and 1000 copy number variants [16] with respect to the genome reference sequence. Around 20,000–25,000 of these variants are protein coding, of which 10,000 change an amino acid but only 50–100 are protein truncating or loss of function variants [16]. Manual review of large numbers of variants is impractical and costly and there are additional difficulties, such as a lack of functional annotation or the interpretation of multiple variants within a haplotype.

Variant interpretation often considers the impact of a variant on a transcript or protein. It is dependent, therefore, on transcript annotation and localizing variants to protein-coding or non-coding regions. There are two major sources of *Homo sapiens* annotation: GENCODE [17] and Reference Sequence (RefSeq) [18] at the National Center for Biotechnology Information (NCBI). Both sets of transcript annotation are subject to version changes and updates that can modify variant reporting and interpretation. For data reproducibility, transcript isoforms and transcript versions must be rigorously tracked, although in some cases even including the version is not sufficient to avoid all potential misinterpretations [19]. There are differences in how the transcript sets are produced: GENCODE annotation is genome-based while RefSeq transcripts are independent of the reference genome. Although RefSeq transcripts may correct for errors in the reference assembly and provide transcripts with improved biological representation (such as for the genes ABO, ACTN3, and ALMS1 in the GRCh37 reference), differences between a genome and a transcript set can cause confusion and errors when reporting variants at the cDNA and genomic levels (e.g., these descriptions refer to the same variant: NM_000059.3:c.7397C>T, NC_000013.11:g.32355250T=). GENCODE’s aim is to create a comprehensive transcript set to represent expression of each isoform across any tissue and stage of development and, as a result, there are, on average, nearly four transcript isoforms per protein-coding gene. Most genes, therefore, have several annotations for a given variant due to multiple transcript isoforms (the G protein-coupled receptor 56 gene (GPR56) in Ensembl release 79 has 61 transcripts). This number will increase as more experimental data accumulate. Choosing the correct transcript isoform and version for consistent variant annotation is challenging. Finally, in loci where the reference genome has several alternative haplotype representations (“ALTs”), variants may have different interpretations with respect to different ALTs. For example, rs150580082 has mappings to multiple ALTs but introduces a stop codon in only some of these. In this case, considering the primary assembly mapping alone will give misleading results.

Variant reporting using Human Genome Variation Society (HGVS) nomenclature is also based on transcripts or proteins. Therefore, the difficulties with transcript annotation described above may cause confusion and ambiguities when using HGVS nomenclature. Many possible annotations exist for variants in genes with multiple transcript isoforms. For example, rs121908462 is a pathogenic variant associated with polymicrogyria that falls in ADGRG1, an adhesion G protein-coupled receptor G1. This variant has 126 HGVS descriptions in Ensembl [20] (and even more valid HGVS descriptions exist), as it overlaps 75 transcripts, and another 103 different descriptions in dbSNP. Multiple transcripts per locus result in greater numbers of annotations. These require filtering in a consistent manner, which increases the instability and complexity of variant interpretation.

Given these analysis challenges and the increasing volume of sequencing data being produced, there is a need for a robust computational tool to aid prioritization of variants across transcripts and manage the complexities of variant analysis. To facilitate this, we developed the Ensembl Variant Effect Predictor (VEP) [21], which differs significantly from other tools [22] (see Table 1 and the “Discussion” section) and from the previously published Ensembl SNP Effect Predictor [23]. The VEP is a software suite that performs annotation and analysis of most types of genomic variation in coding and non-coding regions of the genome. From disease investigation to population studies, it is a critical tool to annotate variants and prioritize a subset for further analysis.

**Table 1:** Comparison of features of VEP with Annovar [95] and SnpEff [66]

Class	Feature	VEP	Annovar	SnpEff
General	Language	Perl	Perl	Java
	Availability (non-commercial)	Free	Registration required	Free
	Availability (commercial)	Free	License required	Free
	Licence	Apache 2.0	Unspecified, not open source	LGPLv3
Input	VCF	Yes	Yes	Yes
	rsID	Yes	No	No
	HGVS	Yes	No	No
	BED	No	No	Yes
	Sequence variants	Yes	Yes	Yes
	Structural variants	Yes	Yes	Yes
Output	VCF	Yes	Yes (non-standard)	Yes
	HGVS	Yes	Yes	Yes
	Summary statistics	Yes	Yes	Yes
	Graphical summary	Yes	No	Yes
	Customizable output	Yes	No	No
Transcript sets	Ensembl	Yes	Yes	Yes
	RefSeq	Yes	Yes	Yes
	GENCODE Basic	Yes	Yes	No
	Species supported	~5000	94	~4500
	User-created databases	Yes	Yes	Yes
Interfaces	Local package	Yes	Yes	Yes
	Submission-based web interface	Ensembl Tools	wAnnovar	Galaxy
	Instant prediction web interface	Yes	No	No
	Cloud/VM	Yes	No	Yes
	API access	Perl, REST	No	No
Consequence types	Sequence Ontology	Yes	No	Yes
	Impact classification	Yes	No	Yes
	Number of classes	33	19	42
	Default reporting level	Transcript	Gene	Transcript
	Summary level reporting	Optional, customisable	Default, customisable	No
	Splicing predictions	Yes (via plugins)	Yes (via external data)	Yes (experimental)
	Loss of function prediction	Yes (via plugins)	No	Yes
	Nonsense mediate decay assessment	No	No	Yes
Non-coding	Regulatory features	Yes	Yes	Yes
	Support multiple cell lines	Yes	No	Yes
	TFBS scoring	Yes	No	No
	miRNA structure location	Yes (via plugins)	No	No
Known variants	Report known variants	Yes	Yes	Yes
	Filter by frequency	Yes	Yes	Yes
	Clinical significance	Yes	Yes	Yes
Other filters	Pre-set filters	Yes	Yes	Yes
	Arbitrary filtering	Yes	No	Yes
Other	Per-individual annotation	Basic	No	Somatic versus germline
	Annotation with custom data	Yes	Yes	Yes
	Custom code extensions via Plugin architecture	Yes	No	No

*miRNA* microRNA, *TFBS* transcription factor binding site, *VM* virtual machine

The VEP has been used for analysis of traits in farm animals [24, 25], for patient diagnosis in the clinic and for research on GWAS [26–30]. It has been used for analysis in numerous large-scale projects, including the 1000 Genomes [31] and Exome Aggregation Consortium (ExAC) [32]. VEP’s annotations are used as input to tools for deep exploration of variant annotation such as GEMINI [33]. It is a flexible tool of value to any project requiring detailed annotation of sequence variants.

## Results

The VEP annotates two broad categories of genomic variant: (1) sequence variants with specific and well-defined changes (including SNVs, insertions, deletions, multiple base pair substitutions, microsatellites, and tandem repeats); and (2) larger structural variants (greater than 50 nucleotides in length), including those with changes in copy number or insertions and deletions of DNA. For all input variants, the VEP returns detailed annotation for effects on transcripts, proteins, and regulatory regions. For known or overlapping variants, allele frequencies and disease or phenotype information is included.

The VEP can be used to analyze data from any species with an assembled genome sequence and an annotated gene set. The data files necessary for annotation in 80 vertebrate species and many invertebrates are distributed by Ensembl and Ensembl Genomes [34], respectively. These are updated regularly, ensuring analysis can be performed using contemporary biological knowledge. The VEP also supports both the latest GRCh38 and previous GRCh37 human assemblies. Importantly, all results are fully reproducible using Ensembl archived versions. Finally, researchers may use their own transcript data for analysis, e.g., in species not yet in Ensembl or for novel or private annotations. A script is included in the VEP script package to create an annotation set from a general feature format (GFF) and FASTA file pair.

Each version of the VEP is tied to a specific release of Ensembl. This explicit versioning ensures all results are stable across a release, which is critical for provenance and reproducibility. To avoid misinterpretation of a variant based on a previous transcript or protein version, the output includes the identifier and version in HGVS coding descriptions. The VEP is open source, free to use, and actively maintained and developed. A mailing list [35] provides responsive support and the benefits of a shared community. The wide usage helps ensure bugs are found and corrected rapidly and enables suggestions to be gathered from a broad range of project teams.

The nature of the VEP results are described below along with input and output formats, the different interfaces, and details on performance.

### Transcript annotation

The VEP results include a wide variety of gene and transcript related information (Table 2). Any transcript set on a primary reference assembly or on ALT sequences can be used but the VEP selects Ensembl annotation by default. For *Homo sapiens* and *Mus musculus* this is the GENCODE gene set, which denotes that it is a full merge of Ensembl’s evidence-based transcript predictions with manual annotation to create the most extensive set of transcript isoforms for these species [36]. The Ensembl transcripts match the reference genome assembly exactly, which eliminates the potential for errors in annotation due to differences between the reference and transcript annotation. If configured to use the RefSeq transcript set, mismatches between a transcript and the genome reference assembly are reported to eliminate possible confusion in the interpretation.

**Table 2:** Gene and transcript-related fields reported by the VEP

Property	Description
Gene ID	Ensembl stable identifier for affected gene
Gene symbol	Common name for gene, e.g., from HGNC
Transcript ID	Ensembl stable identifier for affected transcript
RefSeq ID	NCBI RefSeq identifier for affected transcript
CCDS ID	Consensus coding sequence (CCDS) identifier uniting Havana, Ensembl, and NCBI
Biotype	GENCODE biotype of affected transcript
cDNA coordinates	Coordinates of input variant in unprocessed cDNA
CDS coordinates	Coordinates of input variant in processed coding sequence (CDS)
Distance	Distance to transcript if variant falls outside transcript boundaries
Consequence type	SO consequence type of input variant allele on transcript
Exon	Number(s) of affected exon(s)
Intron	Number(s) of affected intron(s)
TSL	Transcript Support Level (TSL) highlights well-supported and poorly supported transcript models
APPRIS	Annotation principle splice isoforms (APPRIS) is a system to annotate alternatively spliced transcripts based on a range of computational methods, assigning primary and alternative statuses to transcripts
HGVS	HGVS notations for input variant relative to the coding sequence
Phenotype	Flag indicating known association with a phenotype or disease

A variant may have more than one alternative non-reference allele and may overlap more than one transcript or regulatory region. Therefore, to present the most comprehensive annotation the VEP output reports one line (or unit) of annotation per variant alternative allele per genomic feature. As yet, there is no robust annotation of dominant transcript per tissue type available so the VEP includes a variety of data to help filter the many different transcript isoforms. For example, in *H. sapiens* and *M. musculus* the filtered GENCODE Basic transcript set includes the vast majority of transcripts identified as dominantly expressed [36] and consensus coding sequence (CCDS) annotation highlights transcripts having the same CDS in both RefSeq and Ensembl. In several species, a ranking of supporting evidence for transcripts using Transcript Support Level data can prioritize consequences for review [37] while APPRIS provides automated annotation of principal transcript isoforms [38]. Cross-references to known proteins in UniProt and the option to filter for variants in protein coding transcripts are also included. In *H. sapiens*, for clinically relevant loci requiring stable annotation, the VEP can annotate on Locus Reference Genomic (LRG) sequences. Furthermore, the VEP has a flexible “plugin” architecture (described in the “VEP Script” section) to enable for algorithmic extensions additional analysis. For example, an experimental plugin, GXA.pm, uses data from the Expression Atlas project [39] to indicate expression levels across tissues for many transcripts, which can be used to filter transcript isoforms.

### Protein annotation

Protein sequence changes are annotated with the information in Table 3. The VEP also provides an indication of the effect of the amino acid change using protein biophysical properties. These data can improve interpretation of protein variants with no associated phenotype or disease data by predicting how deleterious a given mutation may be on the functional status of the resultant protein. Scores and predictions are pre-calculated for all possible amino acid substitutions and updated when necessary, ensuring that even the annotation of novel variants is rapid. Sorting Intolerant From Tolerant (SIFT) [40] results are available for the ten species that are most used in Ensembl. PolyPhen-2 [41] results are available for human proteins. Other pathogenicity predictor scores such as Condel [42], FATHMM [43], and MutationTaster [44] are available for human data via VEP plugins (Table 4).

**Table 3:** Protein-related fields reported by the VEP

Property	Description
Protein ID	Ensembl stable identifier for affected protein product
RefSeq ID	NCBI RefSeq identifier for affected protein
SWISSPROT ID	Manually curated protein identifier from UniProt
TrEMBL ID	Automatically generated identifier from UniProt
UniParc ID	Combined protein identifier from UniProt
Protein coordinates	Coordinates of input variant in protein product
Codons	Reference and alternative codons as generated by input variant
Amino acids	Reference and alternative amino acids as generated by input variant
SIFT	SIFT pathogenicity prediction and score
PolyPhen	PolyPhen-2 pathogenicity prediction and score
Protein domains	Protein domains overlapping input variant
HGVS	HGVS notations for input variant relative to the protein sequence

**Table 4:** Examples of VEP plugins

Plugin	Maintained by	Functionality
CADD	Martin Kircher	Integrates multiple annotations into one metric by contrasting variants that survived natural selection with simulated mutations
dbNSFP	Ensembl	Provides pre-calculated scores from dbNSFP for many pathogenicity prediction tools for every possible missense variant in the human genome [96]
dbscSNV	Ensembl	Retrieves data for splice variants from dbscSNV [97]
ExAC	Ensembl	Retrieves ExAC allele frequencies from the Exome Aggregation Consortium (ExAC) project [32]
GWAVA	Graham Ritchie	Predicts the functional impact of variants on non-coding elements from, e.g., ENCODE using GWAVA
GXA	Ensembl	Reports data from the Expression Atlas
LD	Ensembl	Finds variants in linkage disequilibrium with any overlapping existing variants
LOFTEE	Konrad Karczewski	Predicts if stop gain, splice site, or frameshift variants lead to loss of function (LoF) in the affected protein
MaxEntScan	Ensembl	Compares scores for reference and mutant splice site sequences using a maximum entropy method
miRNA	Ensembl	Reports whether a variant is predicted to fall in a stem or loop region of a mature miRNA
UpDownStream	Ensembl	By default the VEP searches 5 kb either side of input variants for transcripts. Configures this distance which is useful in species with small intergenic distances or for investigating long-range trans-acting regulatory interactions
VAX	Michael Yourshaw	Incorporates data from KEGG, Human Protein Atlas, MitoCarta, OMIM, and more into VEP output

For a full list of plugins see [76]

### Non-coding annotation

Variants in non-coding regions may have an impact on transcriptional or translational regulation if they fall in regulatory regions. The VEP reports variants in non-coding RNAs, genomic regulatory regions, or transcription factor binding motifs and also reports changes to the consensus score of binding motifs (Table 5), which have been shown to be implicated in disease [45]. The Ensembl Regulatory Build [46], which uses data from ENCODE [47], BLUEPRINT [48], and the NIH Epigenomics Roadmap [49], is the primary regulatory annotation but the VEP analysis can be limited to regulatory regions observed in specific cell types. GERP [50] and other conservation scores derived from genomic multiple alignments, which may predict functional importance in non-coding regions, can be added via a plugin. GWAVA [51], CADD [52], and FATHMM-MKL [53] plugins are also available, which integrate genomic and epigenomic factors to grade and prioritize non-coding variants.

**Table 5:** Regulatory element-related fields reported by the VEP

Property	Description
Regulatory or Motif feature ID	Ensembl identifier for affected regulatory element
Motif name	External name for transcription factor binding motif
Motif position	Coordinates of input variant in transcription factor binding motifs
Motif score	Score reflecting effect of input variant on closeness of binding motif sequence to consensus
Informative position	Flag indicating if the position occupied by the variant in the binding motif is important in the consensus sequence

### Frequency, phenotype, and citation annotation

The VEP searches the Ensembl Variation databases, which contain a large catalogue of freely available germ line and somatic variation data in vertebrates [54, 55]. Ensembl integrates and quality checks variants from dbSNP [56] and other sources for 20 species. Additional human data include mutations from COSMIC [57] and the Human Gene Mutation Database [58] and structural variants and copy number variants from the Database of Genomic Variants archive [59]. Therefore, the VEP can reference millions of variants to identify those previously reported. The VEP reports allele frequencies from the 1000 Genomes, NHLBI exome sequencing [60], and ExAC projects. These can be used as filters, allowing common variants to be excluded as candidates for pathogenicity (see Table 6 for a list of the annotations provided and Table 7 for filters). The VEP includes PubMed identifiers for variants which have been cited and also annotates those associated with a phenotype, disease, or trait using data from OMIM [61], Orphanet [62], the GWAS Catalog [63], and other data sources [64]. Clinical significance states assigned by ClinVar [65] are also available for human variants.

**Table 6:** Co-located variant-related fields reported by the VEP

Property	Description
Variant ID	External identifier for variant co-located with input, e.g., rsID from dbSNP
Somatic	Somatic status of co-located variant
GMAF	Global minor allele and frequency of co-located variant from combined 1000 Genomes phase 3 populations
Other frequencies	Frequency data from continental level 1000 Genomes phase 3 data and two NHLBI–Exome Sequencing Project populations
Clinical significance	Clinical significance status of co-located variant as reported by ClinVar
Phenotype	Flag indicating known association with a phenotype or disease
PubMed ID	NCBI PubMed IDs of publications citing co-located variant

**Table 7:** Example filters available in the VEP

Option or command	Description
Runtime filters	
--no_intergenic	Filter out variants that fall in intergenic regions
--pick	Choose one consequence for each variant; priority is given to the canonical transcript for each gene, protein coding transcripts, and more severe consequence types e.g., missense_variant is more severe than intron_variant
--per_gene	Picks one consequence using the same methodology as --pick but chooses one per overlapping gene
--filter_common	Filter out variants that are co-located with a known variant that has a minor allele frequency greater than 1 %.
Results filters using filter_vep.pl	
SIFT is deleterious OR PolyPhen is probably_damaging	Filter for results where SIFT or PolyPhen-2 predicts the variant protein will be non-functional
AFR >0.1 AND EUR <0.05	Filter for variants co-located with those that are common in African populations but rare in European populations
Gene in gene_list.txt AND Phenotype matches cancer	Filter for results for variants that fall in the genes with IDs listed in gene_list.txt and that have been annotated with a cancer phenotype from a custom dataset (VEP script only)

### Input and output formats

The VEP supports input data in variant call format (VCF), the standard exchange format used in next-generation sequencing pipelines. Unlike other tools (Table 1), the VEP can also process variant identifiers (e.g., from dbSNP) and HGVS nomenclature notations (e.g., HGVS using Ensembl, RefSeq, or LRG transcripts and proteins ‘ENST00000615779.4:c.102944T>C’; ‘BRCA2:p.Val2466Ala’; ‘Q15118:p.Val42Phe’). These identifiers are commonly used in publications and reports. This functionality can also be used to “reverse map” variants from cDNA or protein coordinates to the genome and vice versa.

VEP output consists of an HTML or text format summary file and a primary results file in tab-delimited, VCF, GVF, or JSON format. The default tab-delimited output is designed to present key data in a human-readable format that is easily parsed and can include detailed and complex data alongside. The VEP’s VCF output follows a standard agreed with other annotation tool providers [66] to promote transparent cross-comparison and benchmarking of results.

Variant consequences are described using a standardized set of variant annotation terms [67] which were defined in collaboration with the Sequence Ontology (SO) [68]. Each consequence term has a stable identifier and definition, thereby removing ambiguity in definition or meaning. Structuring the consequences ontologically enables powerful querying: it is possible to retrieve all coding variants in one query without the need to specify each sub-category such as stop_gained, missense, synonymous, etc. The SO terms are used widely, including by the UCSC Genome Browser [69], the 1000 Genomes Project [70], ClinVar, the ExAC project, and the International Cancer Genome Consortium [71], allowing transparent interoperability and cross-validation.

### VEP interfaces

The VEP is platform independent and available as (1) an online tool, (2) an easily installed Perl script, or (3) via the Ensembl Representational State Transfer (REST) application program interface (API) [72]. Each interface is optimized to support different quantities of data and levels of bioinformatics experience. All three use the same core codebase to ensure results are consistent across each interface. A comprehensive test suite backs all code, with continuous integration performed by Travis CI [73], ensuring high quality code, which must pass stringent quality tests before release.

#### VEP Web

VEP Web [21] offers a simple point-and-click interface. This is ideal for exploring annotation in an interactive manner. The portal is most suited to first-time use or small-scale analysis. The maximum compressed uploaded data file size currently supported is 50 megabytes, large enough for around two million typical lines of VCF data.

For single variant analysis, the web interface incorporates ‘Instant VEP’ functionality. Pasting or typing a single variant such as a variant in HGVS notation from a manuscript will rapidly return basic consequence prediction data. To submit a request for more than one variant, data can be uploaded, pasted or given via URL and options selected using a simple online form. A limited set of the VEP’s most commonly used plugins is available to use via the web interface. Requests are processed by a resource management system on the Ensembl web servers to distribute the request load.

**Fig. 1:**
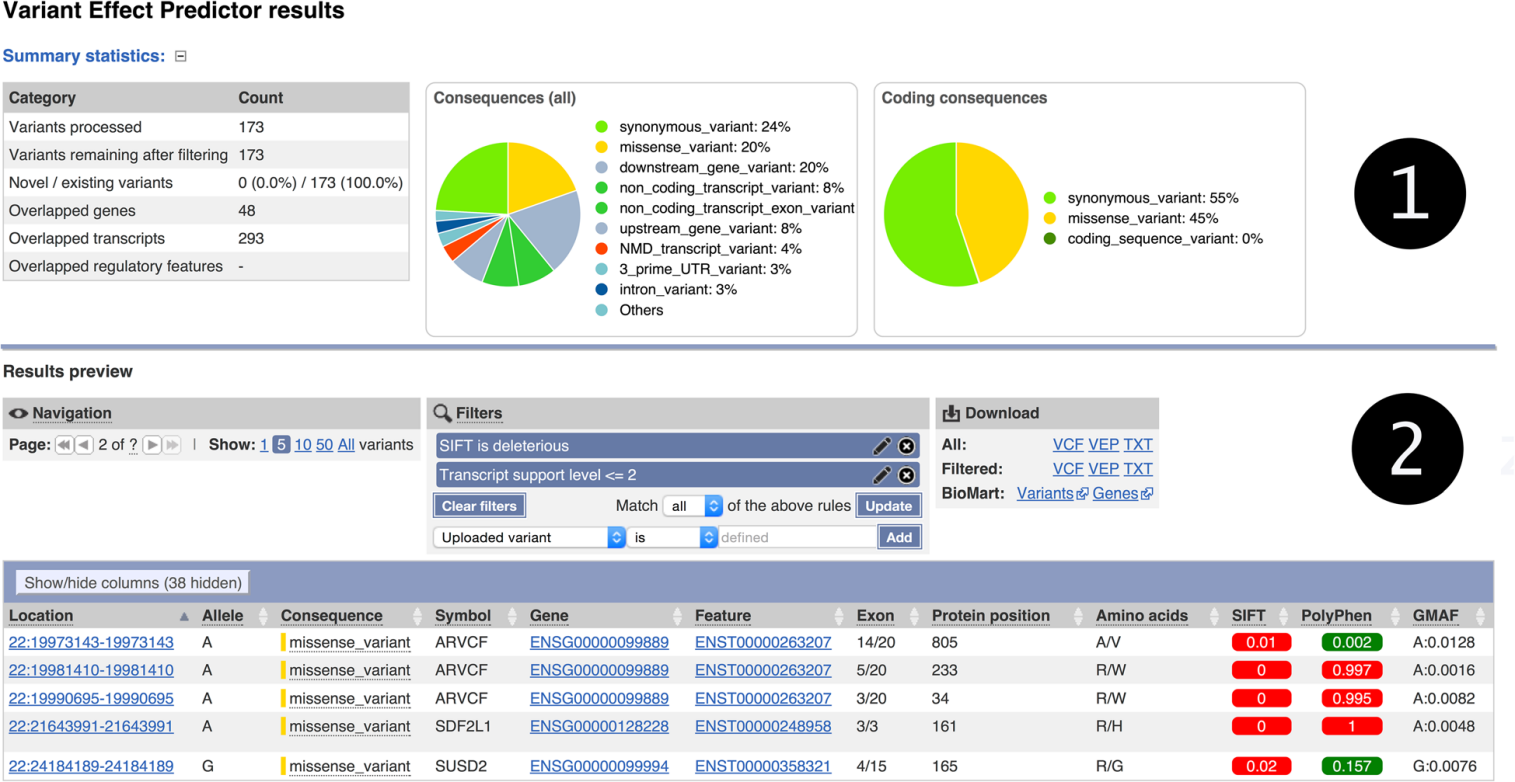
A typical VEP Web results page. Section (1) gives summary pie charts and statistics. Section (2) contains a preview of the results table with navigation, filtering, and download options. The preview table contains hyperlinks to genes, transcripts, regulatory features, and variants in the Ensembl browser. The results can be downloaded in VCF, text, or custom VEP file formats

The output web page (see example in Fig. 1) shows summary statistics and charts to provide an overview of the results. It also has a table with a preview of the detailed results, with a simple interface to configure filtering of the output. Via a series of drop-down menus, multiple filters (see examples in Table 7) can be combined using basic logical relationships, thereby allowing the creation of complex customized queries. This is designed to aid prioritization of smaller numbers of variants. Results can be stored by logging into an Ensembl account.

#### VEP script

The downloadable Perl script [74] is the most powerful and flexible way to use the VEP. It supports more options than the other interfaces, has no limit on input file size, and includes extensive input, output, filtering, and analysis options.

To install the script, simply download the VEP package and run the installer script, which automatically downloads the necessary API and annotation files (or ‘cache’ files). Updates with the latest data are available for each Ensembl release. The full source code is freely available on the Ensembl GitHub repository.

To process large volumes of data, the VEP script works most efficiently in “offline” mode using a local cache of transcript annotations rather than online public databases. As well as optimizing runtime, this ensures data privacy for clinically or commercially sensitive data. Furthermore, the VEP input can be configured to query overlaps with local, potentially private, variant and phenotype data or other custom data sets in a manner similar to vcfanno [75]. In this way annotation in formats including BED, GFF, GTF, VCF, and bigWig can be incorporated into the VEP output.

Advanced filtering options are available for a smaller result set, either during runtime or as a post-run process (Table 7). Filtering can be performed as a post-run process by an accompanying script that uses a simple field-operator-value language. Filtered results can be fed back to the VEP for further analysis or exported.

With some familiarity of Perl, the VEP can truly be customized, extended, and integrated with other systems. As almost all of the algorithmic content of the VEP is contained within the Ensembl API, the features of the VEP can be accessed using API calls. It is trivial, therefore, to extend the VEP results and perform secondary analyses, such as retrieving all OMIM IDs associated with the genes in the VEP results or calculating known variants in linkage disequilibrium with a subset of variants. Alternatively, the VEP is also customizable via its plugin architecture, which was developed to provide greater scope for expansion. This architecture supports the use of VEP as the backbone of a customized analysis pipeline by writing additional code to extend the VEP’s functionality for specific use cases. Example uses include filtering output, adding annotation from local or remote sources, executing external programs, or rendering graphical representations of the output. Ensembl provides a number of VEP plugins, hosted on GitHub [76], and a variety are published [51, 77] (Table 4).

#### VEP REST API

**Fig. 2:**
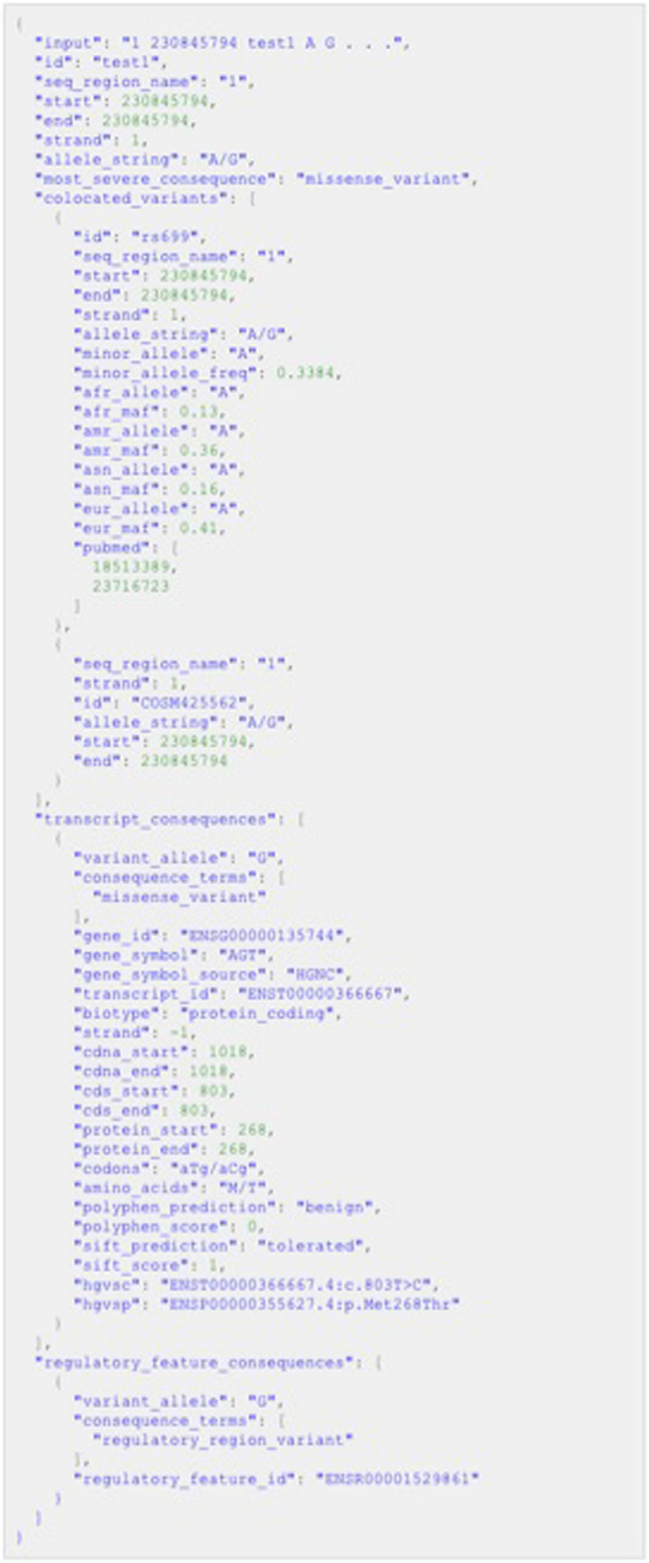
Example of JSON output as produced by the VEP script and REST API (redacted and prettified for display)

Ensembl’s language-independent REST API provides robust computational access in any programming language and returns basic variant annotation and consequence data. Individually or in batches of up to 1000, variants can be submitted to the API server in a single request. Results return in JSON, simple for parsing in most modern programming languages (see Fig. 2 for an example of JSON output). Using this interface, dynamic VEP queries can be integrated into custom-built software for on-demand results, as used, e.g., in the Decipher Genome Browser [78]. For documentation see [79].

As with the web interface, a limited set of the VEP’s most commonly used plugins is configured for use via the REST API.

## Discussion

### Performance

The VEP script can be threaded for rapid performance on systems with multiple CPU cores. A typical human individual’s variant set can be processed in around an hour on a modern quad core machine; the 4,474,140 variants in NA12878 from Illumina’s Platinum Genomes set [80] took 62 minutes to process (Table 8). This reduces to 32 minutes using the smaller GENCODE basic gene set. A negligible startup time means the VEP achieves similar throughput rates on both small and large datasets. A typical exome sequencing data set (100,000 to 200,000 variants) is processed in under 5 minutes.

**Table 8:** Comparison of runtime

Tool	Chr. 21	All
Annovar	0 m38.933 s (1732 v/s)	21 m50.037 s (3415 v/s)
SnpEff	1 m46.178 s (635 v/s)	46 m39.142 s (1598 v/s)
SnpEff (threaded)*	1 m21.046 s (832 v/s)	10 m28.274 s (7121 v/s)
VEP	0 m47.216 s (1428 v/s)	62 m9.107 s (1200 v/s)

Two datasets from Illumina’s Platinum Genomes were used [93], both on the GRCh37 assembly: 67416 variants from chromosome 21 and the whole genome set of 4,474,140 variants. Each tool was configured to use the Ensembl release 75 gene set, with options configured for the fastest runtime. Run time and speed in variants per second (*v/s*) are shown. *SnpEff was run in threaded mode but multiple warnings and errors were produced during these runs.

To improve runtime, individual VEP jobs can be threaded across multiple processor cores. Larger scale parallel processing architectures such as compute farms enable further subdivision of the VEP job (for example, by chromosome).

The VEP’s runtime performance is compared with Annovar and SnpEff in Table 8. For smaller input files, the VEP performs as well as or faster than other tools. The VEP concedes time to SnpEff by being written in Perl (an interpreted language) versus compiled Java for SnpEff [81]. SnpEff loads its entire annotation database into memory at start-up, unlike VEP, which loads the relevant genomic segments on demand; this accounts for VEP performing better than SnpEff on smaller datasets. Annovar, while also written in Perl, does not provide the same depth of annotation as VEP and so runs faster. It should also be noted that the VEP, through the REST API or through the Instant VEP functionality of the VEP web interface, returns predictions for single variants in a fraction of a second. This is available to users without any software download or installation, something neither Annovar nor SnpEff can offer.

Run time varies with the number and complexity of overlapping genomic features, resulting in faster analysis times for species with sparse annotation than those with rich annotation such as human and mouse.

As the web and REST implementations are based on the same underlying code as the VEP script, performance is broadly comparable to the above, with allowances made for job queues (for web), network transfer of data (for web and REST), and request limits (for REST).

## Conclusions

The Ensembl Variant Effect Predictor software provides tools and methods for a systematic approach to annotate and prioritize variants in both large-scale sequencing projects and smaller analysis studies. By automating annotation in a standard manner and reducing the time required for manual review, it helps manage many of the common challenges associated with analysis of SNVs, short insertions–deletions, copy number variants, and structural variants. The VEP annotates variants using a wide range of reference data, including transcripts, regulatory regions, frequencies from previously observed variants, citations, clinical significance information, and predictions of biophysical consequences of variants.

The quality, quantity, and stability of variant annotation obtained depends on the choice of transcript set used [82]. As such, the VEP allows flexibility of transcript choice. To effectively manage large numbers of variant annotations and transcript isoforms, the VEP provides several methods to prioritize results and reduce the number of variants needing manual review. A selection of these filters is available and VEP also supports building of custom filters. Uniquely, the VEP algorithm can be expanded to perform additional calculations via plugins [77] and can analyze custom, potentially private, data.

Interpreting all variants in a genome remains an unsolved challenge. An increasing number of large-scale WGS will detect rare variants in both coding and non-coding regions of the genome and further possible identification of loci associated with disease. Having these variants available in public repositories such as dbSNP and the European Variant Archive or discoverable using federated resources will be of significant benefit for analysis. Emerging efforts such as the Global Alliance for Genomic Health (GA4GH) Beacon project [83] are currently developing possible distributed solutions.

Improved functional annotation is especially critical for variants in non-coding regions. Many fall in loci that regulate gene expression in specific tissues. Characterizing associations between transcripts and tissues will facilitate a subset of tissue-specific transcript isoforms to be selected for variant annotation, tailoring results. Moreover, upon providing the link from regulatory region to regulated gene, the potential molecular mechanism underlying disease could be explained. Data from large scale efforts such as the Genotype-Tissue Expression project, which aims to systematically characterize the effects of regulatory variants in different tissues [84], will be integrated into the VEP reference data in order to have the most current data available to the VEP for analysis.

As discussed above, standardized SO terms are used for describing variant consequences and VEP results can be output in VCF format. Work is ongoing to develop a comprehensive variant annotation data exchange format within the GA4GH. Furthermore, the GA4GH is defining standards for representation of associations between variants and phenotypes, traits, and diseases. The VEP will support such formats when they are mature.

Current annotation tools, including the VEP, annotate each input variant independently, without considering the potential compound effects of combining alternate alleles across multiple variant loci. This limitation means that having two or more variants affecting the same codon, or a shift in reading frame being corrected by a downstream variant, will not be taken into consideration. In future, given genotype data phased into haplotypes, the VEP will accurately annotate such events.

The VEP is also regularly extended and improved (see release notes at [85]) with new features added to both the core VEP code and the plugin library. Although these developments are frequently driven by new annotations or datasets available for *H. sapiens*, they are all designed to be compatible with any species. Once additional annotation and sequencing data are available in other species, the VEP extensions can be fully exploited for these too (e.g., 1000 Bulls project, the 1000 Chicken project, the 1001 Arabidopsis project, and the Functional Annotation of ANimal Genomes (FAANG) consortium). To improve genome-wide analysis, the VEP will leverage data from future sequencing projects, implement new algorithms and adopt data exchange standards and, therefore, bring continual benefit to variant interpretation.

## Methods

The VEP algorithms and code are part of the freely available Ensembl API, coded in the Perl programming language. Time-critical components are written in C and incorporated into the API using the XS framework [86]. Installation of the VEP script triggers automated installation of the Ensembl API, along with the BioPerl API [87] upon which the Ensembl API depends. All interfaces to the VEP use the same underlying API calls, ensuring consistency across the different VEP access platforms when version control is observed.

To process the input data, sequential contiguous blocks of variants (default block size 5000) are read into an input memory buffer. Each variant is converted into an Ensembl VariationFeature object that represents a genomic location and a set of alleles. Variants in tab-delimited and Pileup formats are converted directly to objects; those in HGVS notation are resolved to their genomic coordinates by extracting the relevant reference feature (transcript, protein, or chromosome) using the Ensembl API. VCF input undergoes pre-processing to account for differences in how VCF and Ensembl represent unbalanced substitutions and indels. When using VEP’s forking functionality, the input buffer is divided amongst a number of sub-processes. Each sub-process carries out the analysis described hence and then the results are rejoined and sorted back into input order before being written to output.

Normalization of insertions and deletions in repetitive sequence and decomposition of complex variants are recommended as part of a robust pipeline to ensure consistency of annotation across datasets. Optionally, in a process analogous to that described in [88], VEP’s parser can be forced to decompose alternative alleles in complex variant descriptions to their most minimal representation by stripping identical bases from the 5′ and 3′ ends of the reference and alternative allele. This is not done by default as it may change the input position and allele string provided. Similarly, although it is a recommendation of the VCF format, the VEP does not left-normalize insertion or deletion variants in repetitive sequence. Enforcing this by default would cause discrepancies in input and output coordinates and also for HGVS nomenclature, whose coordinates must be right-normalized with respect to the transcript sequence. Tools such as vt [88] can be used to pre-process VCF input before use in VEP.

Input variants pass through a configurable quality-control process that checks for irregularities and inconsistencies. Variants that fail are reported via standard error output and/or in a warnings file. Checks include, for example, that allele lengths match input coordinates and the input reference allele matches that recorded in the reference genome.

The genomic loci overlapped by the variants in the input buffer are resolved to distinct megabase-sized regions. Each region corresponds to a single file on disk in the VEP cache, which contains objects serialized using Perl’s Storable framework [89]. For each region, the transcripts, regulatory features, and known variants are loaded from disk, deserialized into objects, and cached in memory. This avoids rereading from disk when the same region is overlapped by variants in consecutive input buffers. The publicly available Ensembl databases can be used in place of the cache files to avoid downloading the data in advance, though doing so incurs a performance penalty due to network transfer rates.

Transcripts have a configurable flank (default 5000 base pairs) to allow the VEP to assign upstream and downstream status to variants within the region flanking a transcript. A hash-based tree structure is used to search for overlaps between input variants and genomic features. For each overlap, a VariationFeatureOverlap object is created, with specific sub-classes for each genomic feature type: TranscriptVariation, RegulatoryFeatureVariation, MotifFeatureVariation. Each VariationFeatureOverlap object has two or more child VariationFeatureOverlapAllele objects representing each allele of the input variant—one representing the reference allele and one or more representing each of the alternative or mutant alleles. These objects are also sub-classed, with, for example, a TranscriptVariationAllele representing one allele of a variant overlapping a Transcript object.

For each TranscriptVariationAllele object, the API evaluates consequence types using a set of predicate functions. These assess whether, for example, a variant is predicted to cause a change in protein coding sequence (e.g., missense_variant). Prior to this, a series of pre-predicate checks are performed to improve runtime; for example, a variant does not need to be assessed for change to the protein sequence if it falls entirely within the intron of a transcript. These pre-predicate checks are also cached at each object “level”; for example, the location of a variant relative to the transcript structure is fixed at the TranscriptVariation level but the allele type can be different for each TranscriptVariationAllele. The pre-predicate checks improve runtime by a factor of around two on a typical resequencing-based input file. Without them, runtime is proportional to *nfp*, where *n* is the number of input variant alleles, *f* is the number of overlapped features, and *p* is the number of predicates; depending on a number of factors this can become as low as *nfp*/2 with pre-predicate checks enabled.

Predicates also make extensive use of caching: UTR, coding, and translated sequences are all cached on the Transcript object with intron structure and other frequently accessed data. Established components of the Ensembl API handle tasks such as splicing exons and re-translating mutated sequences. Alternative codon tables are used as appropriate for mitochondrial sequences and selenocysteines. If a predicate is true for a given TranscriptVariationAllele, an OverlapConsequence object is assigned representing the consequence type; this object contains the appropriate SO term along with synonyms and ranking information. Each OverlapConsequence object type corresponds to one predicate. Hierarchy in the predicate system preserves the tree structure of the SO such that only the most specific term that applies under any given parent term is assigned; this same tree structure allows for ontological-style querying and filtering of the results. Multiple OverlapConsequence objects may be added to a single VariationFeatureOverlapAllele or TranscriptVariationAllele object to allow for complex cases, such as a variant that falls in a splice-relevant region that also affects the coding sequence of the transcript.

HGVS notations are also derived from TranscriptVariationAlleles, though they undergo significant additional processing to conform to the nomenclature definition [90]. For example, insertions or deletions with respect to the transcript sequence must be reported at the most 3′ position possible when they fall in repetitive sequence.

VariationFeatureOverlapAllele objects are then converted for writing to output, a process that involves several extra stages. VariationFeatureOverlapAlleles can be filtered in various ways which can be configured, for example: reporting only one VariationFeatureOverlapAllele per input variant; removing intergenic VariationFeatureOverlapAlleles (i.e., those produced from variants that don’t overlap a genomic feature); filtering based on allele frequency of a co-located known variant. Additional data fields are retrieved at this stage from relevant objects, for example: external identifiers for transcripts (UniProt, CCDS); exon and intron numbers; clinical significance for co-located variants. It is also at this stage that any configured plugins are executed. They are passed the VariationFeatureOverlapAllele object, which has accessor methods for other objects, e.g., the Transcript, VariationFeature, or genomic Slice. As plugin modules are executed after the VEP consequence calculation, they have access to the VEP and Ensembl API objects before output data are written and return a data structure that is incorporated alongside the VEP’s main output data structure. The output data structure is then written to disk as one of several formats (tab-delimited, VCF, GVF, JSON), with the fields for each data format configurable at runtime. Output files contain headers describing the format and content of data fields, as well as version information for resources used.

### Cache and sequence files

The VEP’s caches are built for each of Ensembl’s primary species (70 species as of Ensembl version 84); the files are updated in concert with Ensembl’s release cycle, ensuring access to the latest annotation data. Cache files for all previous releases remain available on Ensembl’s FTP archive site [91] to facilitate reproducibility. For 15 of these species there are three types of cache files: one with the Ensembl transcripts, a “refseq” one with the RefSeq transcripts, and a “merged” one that contains both. Caches for both the latest GRCh38 and previous GRCh37 (hg19) human genome builds are maintained. The human GRCh38 cache file is around 5 gigabytes in size, including transcript, regulatory, and variant annotations as well as pathogenicity algorithm predictions. Performance using the cache is substantially faster than using the database; analyzing a small VCF file of 175 variants takes 5 seconds using the cache versus 40 seconds using the public Ensembl variation database over a local network (performance can be expected to be slower when using a remote database connection).

The VEP can use FASTA format files of genomic sequence for sequence retrieval. This functionality is needed to generate HGVS notations and to quality check input variants against the reference genome. The VEP uses either an htslib-based indexer [92] or BioPerl’s FASTA DB interface to provide fast random access to a whole genome FASTA file. Sequence may alternatively be retrieved from an Ensembl core database, with corresponding performance penalties.

Cache and FASTA files are automatically downloaded and set up using the VEP package’s installer script, which utilizes checksums to ensure the integrity of downloaded files. The installer script can also download plugins by consulting a registry. The VEP package also includes a script, gtf2vep.pl, to build custom cache files. This requires a local GFF or general transfer format (GTF) file that describes transcript structures and a FASTA file of the genomic sequence.

## References

[1] Eisenstein M (2014). Personalized medicine: Special treatment. Nature..

[2] Weil MK, Chen A (2011). PARP inhibitor treatment in ovarian and breast cancer. Curr Probl Cancer..

[3] The Deciphering Developmental Disorders Study (2015). Large-scale discovery of novel genetic causes of developmental disorders. Nature.

[4] World Health Organisation. Non-communicable diseases: fact sheet. Jan 2015. http://www.who.int/mediacentre/factsheets/fs355/en/. Accessed 17 Mar 2016.

[5] Visscher PM, Brown MA, McCarthy MI, Yang J (2012). Five years of GWAS discovery. Am J Hum Genet..

[6] Saint Pierre A, Génin E (2014). How important are rare variants in common disease?. Brief Funct Genomics.

[7] Zuk O, Schaffner SF, Samocha K, Do R, Hechter E, Kathiresan S (2014). Searching for missing heritability: designing rare variant association studies. Proc Natl Acad Sci U S A..

[8] Hindorff LA, Sethupathy P, Junkins HA, Ramos EM, Mehta JP, Collins FS (2009). Potential etiologic and functional implications of genome-wide association loci for human diseases and traits. Proc Natl Acad Sci U S A..

[9] Puente XS, Beà S, Valdés-Mas R, Villamor N, Gutiérrez-Abril J, Martín-Subero JI (2015). Non-coding recurrent mutations in chronic lymphocytic leukaemia. Nature..

[10] Gudbjartsson DF, Helgason H, Gudjonsson SA, Zink F, Oddson A, Gylfason A (2015). Large-scale whole-genome sequencing of the Icelandic population. Nat Genet..

[11] NHS. NHS set to deliver world-leading genomics project in fight against cancer and rare diseases. http://www.england.nhs.uk/2014/12/22/genomics-project/. Accessed 17 Mar 2016.

[12] Collins FS, Varmus H (2015). A new initiative on precision medicine. N Engl J Med..

[13] Koepfli K-P, Paten B, O’Brien SJ (2015). The Genome 10 K Project: a way forward. Annu Rev Anim Biosci..

[14] Cao J, Schneeberger K, Ossowski S, Günther T, Bender S, Fitz J (2011). Whole-genome sequencing of multiple Arabidopsis thaliana populations. Nat Genet..

[15] Daetwyler HD, Capitan A, Pausch H, Stothard P, van Binsbergen R, Brøndum RF (2014). Whole-genome sequencing of 234 bulls facilitates mapping of monogenic and complex traits in cattle. Nat Genet..

[16] Gonzaga-Jauregui C, Lupski JR, Gibbs RA (2012). Human genome sequencing in health and disease. Annu Rev Med..

[17] Harrow J, Frankish A, Gonzalez JM, Tapanari E, Diekhans M, Kokocinski F (2012). GENCODE: the reference human genome annotation for the ENCODE Project. Genome Res..

[18] Pruitt KD, Brown GR, Hiatt SM, Thibaud-Nissen F, Astashyn A, Ermolaeva O (2014). RefSeq: an update on mammalian reference sequences. Nucleic Acids Res..

[19] Dalgleish R, Flicek P, Cunningham F, Astashyn A, Tully RE, Proctor G (2010). Locus Reference Genomic sequences: an improved basis for describing human DNA variants. Genome Med..

[20] Cunningham F, Amode MR, Barrell D, Beal K, Billis K, Brent S (2015). Ensembl 2015. Nucleic Acids Res..

[21] Ensembl Variant Effect Predictor web interface. http://www.ensembl.org/vep. Accessed 17 Mar 2016.

[22] Pabinger S, Dander A, Fischer M, Snajder R, Sperk M, Efremova M (2014). A survey of tools for variant analysis of next-generation genome sequencing data. Brief Bioinform..

[23] McLaren W, Pritchard B, Rios D, Chen Y, Flicek P, Cunningham F (2010). Deriving the consequences of genomic variants with the Ensembl API and SNP Effect Predictor. Bioinforma Oxf Engl..

[24] Höglund JK, Sahana G, Brøndum RF, Guldbrandtsen B, Buitenhuis B, Lund MS (2014). Fine mapping QTL for female fertility on BTA04 and BTA13 in dairy cattle using HD SNP and sequence data. BMC Genomics..

[25] Godoy TF, Moreira GCM, Boschiero C, Gheyas AA, Gasparin G, Paduan M (2015). SNP and INDEL detection in a QTL region on chicken chromosome 2 associated with muscle deposition. Anim Genet..

[26] Leslie EJ, Taub MA, Liu H, Steinberg KM, Koboldt DC, Zhang Q (2015). Identification of functional variants for cleft lip with or without cleft palate in or near PAX7, FGFR2, and NOG by targeted sequencing of GWAS loci. Am J Hum Genet..

[27] Hou L, Zhao H (2013). A review of post-GWAS prioritization approaches. Front Genet..

[28] International Multiple Sclerosis Genetics Consortium (2013). Analysis of immune-related loci identifies 48 new susceptibility variants for multiple sclerosis. Nat Genet.

[29] Saunders CJ, Miller NA, Soden SE, Dinwiddie DL, Noll A, Alnadi NA (2012). Rapid whole-genome sequencing for genetic disease diagnosis in neonatal intensive care units. Sci Transl Med.

[30] Wright CF, Fitzgerald TW, Jones WD, Clayton S, McRae JF, van Kogelenberg M (2015). Genetic diagnosis of developmental disorders in the DDD study: a scalable analysis of genome-wide research data. Lancet..

[31] McVean GA, Auton A, Brooks LD, DePristo MA, Durbin RM, Handsaker RE (2012). An integrated map of genetic variation from 1,092 human genomes. Nature..

[32] Exome Aggregation Consortium (ExAC). http://exac.broadinstitute.org. Accessed 17 Mar 2016.

[33] Paila U, Chapman BA, Kirchner R, Quinlan AR (2013). GEMINI: integrative exploration of genetic variation and genome annotations. PLoS Comput Biol..

[34] Kersey PJ, Allen JE, Christensen M, Davis P, Falin LJ, Grabmueller C (2014). Ensembl Genomes 2013: scaling up access to genome-wide data. Nucleic Acids Res..

[35] Developers mailing list. http://lists.ensembl.org/mailman/listinfo/dev. Accessed 17 Mar 2016.

[36] Frankish A, Uszczynska B, Ritchie GR, Gonzalez JM, Pervouchine D, Petryszak R (2015). Comparison of GENCODE and RefSeq gene annotation and the impact of reference geneset on variant effect prediction. BMC Genomics.

[37] Transcript Supporting Level (TSL). http://www.ensembl.org/Help/Glossary?id=492. Accessed 17 Mar 2016.

[38] Rodriguez JM, Maietta P, Ezkurdia I, Pietrelli A, Wesselink J-J, Lopez G (2013). APPRIS: annotation of principal and alternative splice isoforms. Nucleic Acids Res..

[39] Petryszak R, Burdett T, Fiorelli B, Fonseca NA, Gonzalez-Porta M, Hastings E (2014). Expression Atlas update—a database of gene and transcript expression from microarray- and sequencing-based functional genomics experiments. Nucleic Acids Res..

[40] Kumar P, Henikoff S, Ng PC (2009). Predicting the effects of coding non-synonymous variants on protein function using the SIFT algorithm. Nat Protoc..

[41] Adzhubei IA, Schmidt S, Peshkin L, Ramensky VE, Gerasimova A, Bork P (2010). A method and server for predicting damaging missense mutations. Nat Methods..

[42] Gonzalez-Perez A, Deu-Pons J, Lopez-Bigas N (2012). Improving the prediction of the functional impact of cancer mutations by baseline tolerance transformation. Genome Med..

[43] Shihab HA, Gough J, Cooper DN, Stenson PD, Barker GLA, Edwards KJ (2013). Predicting the functional, molecular, and phenotypic consequences of amino acid substitutions using hidden Markov models. Hum Mutat..

[44] Schwarz JM, Cooper DN, Schuelke M, Seelow D (2014). MutationTaster2: mutation prediction for the deep-sequencing age. Nat Methods..

[45] Ward LD, Kellis M (2012). Interpreting noncoding genetic variation in complex traits and human disease. Nat Biotechnol..

[46] Zerbino DR, Wilder SP, Johnson N, Juettemann T, Flicek PR (2015). The Ensembl Regulatory Build. Genome Biol..

[47] The ENCODE Project Consortium (2012). An integrated encyclopedia of DNA elements in the human genome. Nature.

[48] Adams D, Altucci L, Antonarakis SE, Ballesteros J, Beck S, Bird A (2012). BLUEPRINT to decode the epigenetic signature written in blood. Nat Biotechnol..

[49] Romanoski CE, Glass CK, Stunnenberg HG, Wilson L, Almouzni G (2015). Epigenomics: Roadmap for regulation. Nature..

[50] Cooper GM, Stone EA, Asimenos G, Green ED, Batzoglou S, Sidow A (2005). Distribution and intensity of constraint in mammalian genomic sequence. Genome Res..

[51] Ritchie GRS, Dunham I, Zeggini E, Flicek P (2014). Functional annotation of noncoding sequence variants. Nat Methods..

[52] Kircher M, Witten DM, Jain P, O’Roak BJ, Cooper GM, Shendure J (2014). A general framework for estimating the relative pathogenicity of human genetic variants. Nat Genet..

[53] Shihab HA, Gough J, Mort M, Cooper DN, Day INM, Gaunt TR (2014). Ranking non-synonymous single nucleotide polymorphisms based on disease concepts. Hum Genomics..

[54] Chen Y, Cunningham F, Rios D, McLaren WM, Smith J, Pritchard B (2010). Ensembl variation resources. BMC Genomics..

[55] Rios D, McLaren WM, Chen Y, Birney E, Stabenau A, Flicek P (2010). A database and API for variation, dense genotyping and resequencing data. BMC Bioinformatics..

[56] Sherry ST, Ward MH, Kholodov M, Baker J, Phan L, Smigielski EM (2001). dbSNP: the NCBI database of genetic variation. Nucleic Acids Res.

[57] Forbes SA, Bindal N, Bamford S, Cole C, Kok CY, Beare D (2011). COSMIC: mining complete cancer genomes in the Catalogue of Somatic Mutations in Cancer. Nucleic Acids Res..

[58] Stenson PD, Ball EV, Mort M, Phillips AD, Shaw K, Cooper DN. The Human Gene Mutation Database (HGMD) and its exploitation in the fields of personalized genomics and molecular evolution. Curr. Protoc. Bioinformatics. 2012;Chapter 1:Unit1.13. 10.1002/0471250953.bi0113s39 22948725

[59] Lappalainen I, Lopez J, Skipper L, Hefferon T, Spalding JD, Garner J (2013). dbVar and DGVa: public archives for genomic structural variation. Nucleic Acids Res.

[60] NHLBI exome sequencing. http://evs.gs.washington.edu/EVS/. Accessed 17 Mar 2016.

[61] OMIM. http://omim.org/. Accessed 17 Mar 2016.

[62] Orphanet. http://www.orpha.net/. Accessed 17 Mar 2016.

[63] Welter D, MacArthur J, Morales J, Burdett T, Hall P, Junkins H (2013). The NHGRI GWAS Catalog, a curated resource of SNP-trait associations. Nucleic Acids Res..

[64] Ensembl Variation sources of phenotype data. http://www.ensembl.org/info/genome/variation/sources_phenotype_documentation.html. Accessed 17 Mar 2016.

[65] Landrum MJ, Lee JM, Riley GR, Jang W, Rubinstein WS, Church DM (2014). ClinVar: public archive of relationships among sequence variation and human phenotype. Nucleic Acids Res..

[66] Cingolani P, Platts A, Wang LL, Coon M, Nguyen T, Wang L (2012). A program for annotating and predicting the effects of single nucleotide polymorphisms, SnpEff: SNPs in the genome of Drosophila melanogaster strain w1118; iso-2; iso-3. Fly (Austin).

[67] Sequence Ontology terms for describing variant consequences. http://www.ensembl.org/info/genome/variation/predicted_data.html#consequences. Accessed 17 Mar 2016.

[68] Cunningham F, Moore B, Ruiz-Schultz N, Ritchie GR, Eilbeck K. Improving the Sequence Ontology terminology for genomic variant annotation. J Biomed Semant. 2015;6:32. 10.1186/s13326-015-0030-4 PMC4520272 26229585

[69] Rosenbloom KR, Armstrong J, Barber GP, Casper J, Clawson H, Diekhans M, et al. The UCSC Genome Browser database: 2015 update. Nucleic Acids Res. 2014;gku1177. 10.1093/nar/gku1177 PMC4383971 25428374

[70] Clarke L, Zheng-Bradley X, Smith R, Kulesha E, Xiao C, Toneva I (2012). The 1000 Genomes Project: data management and community access. Nat Methods..

[71] The International Cancer Genome Consortium Mutation Pathways and Consequences Subgroup of the Bioinformatics Analyses Working Group. Computational approaches to identify functional genetic variants in cancer genomes. Nat Methods. 2013;10:723–9. 10.1038/nmeth.2562 PMC3919555 23900255

[72] Yates A, Beal K, Keenan S, McLaren W, Pignatelli M, Ritchie GRS, et al. The Ensembl REST API: Ensembl Data for Any Language. Bioinformatics. 2014;btu613. 10.1093/bioinformatics/btu613 PMC4271150 25236461

[73] Travis CI. https://travis-ci.org/. Accessed 17 Mar 2016.

[74] Ensembl Variant Effect Predictor script. http://www.ensembl.org/info/docs/tools/vep/script/index.html. Accessed 17 Mar 2016.

[75] Pedersen BS, Layer RM, Quinlan AR. Vcfanno: fast, flexible annotation of genetic variants. Genome Biol. 2016; 17:118 10.1186/s13059-016-0973-5 PMC4888505 27250555

[76] Ensembl Variant Effect Predictor plugins. https://github.com/ensembl-variation/VEP_plugins. Accessed 17 Mar 2016.

[77] Yourshaw M, Taylor SP, Rao AR, Martín MG, Nelson SF. Rich annotation of DNA sequencing variants by leveraging the Ensembl Variant Effect Predictor with plugins. Brief Bioinform. 2014;bbu008. 10.1093/bib/bbu008 PMC6283364 24626529

[78] Bragin E, Chatzimichali EA, Wright CF, Hurles ME, Firth HV, Bevan AP (2014). DECIPHER: database for the interpretation of phenotype-linked plausibly pathogenic sequence and copy-number variation. Nucleic Acids Res..

[79] Ensembl Variant Effect Predictor REST API documentation. http://rest.ensembl.org/#VEP. Accessed 17 Mar 2016.

[80] Illumina’s Platinum Genomes set. ftp://ussd-ftp.illumina.com/hg19/2.0.1/NA12878/. Accessed 17 Mar 2016.

[81] Differences between compiled and interpreted languages. http://www.codeproject.com/Articles/696764/Differences-between-compiled-and-Interpreted-Langu. Accessed 17 Mar 2016.

[82] McCarthy DJ, Humburg P, Kanapin A, Rivas MA, Gaulton K, Cazier J-B (2014). Choice of transcripts and software has a large effect on variant annotation. Genome Med..

[83] Global Alliance for Genomic Health (GA4GH) Beacon project. https://beacon-network.org/. Accessed 17 Mar 2016.

[84] Ardlie KG, Deluca DS, Segrè AV, Sullivan TJ, Young TR, GTEx Consortium T (2015). The Genotype-Tissue Expression (GTEx) pilot analysis: Multitissue gene regulation in humans. Science..

[85] Ensembl Variant Effect Predictor historical release notes. http://www.ensembl.org/info/docs/tools/vep/script/vep_download.html#history. Accessed 17 Mar 2016.

[86] XS framework. http://perldoc.perl.org/perlxs.html. Accessed 17 Mar 2016.

[87] Stajich JE, Block D, Boulez K, Brenner SE, Chervitz SA, Dagdigian C (2002). The Bioperl toolkit: Perl modules for the life sciences. Genome Res..

[88] Tan A, Abecasis GR, Kang HM (2015). Unified representation of genetic variants. Bioinformatics..

[89] Perl’s Storable framework. http://perldoc.perl.org/Storable.html. Accessed 17 Mar 2016.

[90] den Dunnen JT, Antonarakis SE (2000). Mutation nomenclature extensions and suggestions to describe complex mutations: a discussion. Hum Mutat..

[91] Ensembl’s FTP archive site. ftp://ftp.ensembl.org/pub/. Accessed 17 Mar 2016.

[92] htslib-based indexer. http://www.htslib.org/. Accessed 17 Mar 2016.

[93] Illumina’s Platinum Genomes. http://www.illumina.com/platinumgenomes/. Accessed 17 Mar 2016.

[94] Prebuilt Variant Effect Predictor datasets. ftp://ftp.ensembl.org/pub/current_variation/VEP/. Accessed 17 Mar 2016.

[95] Wang K, Li M, Hakonarson H (2010). ANNOVAR: functional annotation of genetic variants from high-throughput sequencing data. Nucleic Acids Res..

[96] Liu X, Jian X, Boerwinkle E (2013). dbNSFP v2.0: a database of human non-synonymous SNVs and their functional predictions and annotations. Hum Mutat.

[97] Jian X, Boerwinkle E, Liu X (2014). In silico prediction of splice-altering single nucleotide variants in the human genome. Nucleic Acids Res..

